# Between-day reliability of electromechanical delay of selected neck muscles during performance of maximal isometric efforts

**DOI:** 10.1186/1758-2555-1-22

**Published:** 2009-09-23

**Authors:** Sivan Almosnino, Lucie Pelland, Samuel V Pedlow, Joan M Stevenson

**Affiliations:** 1 Biomechanics and Ergonomics Laboratory, School of Kinesiology and Health Studies, Queen's University, Kingston, ON, Canada; 2 School of Rehabilitation Therapy, Queen's University, Kingston, ON, Canada; 3 Human Mobility Research Centre, Kingston General Hospital, Kingston, ON, Canada

## Abstract

**Background:**

The purpose of this study was to assess the between-day reliability of the electromechanical delay (EMD) of selected neck muscles during the performance of maximal isometric contractions in five different directions.

**Methods:**

Twenty-one physically active males participated in two testing sessions separated by seven to eight days. Using a custom-made fixed frame dynamometer, cervical force and surface electromyography (EMG) were recorded bilaterally from the splenius capitis, upper trapezius and sternocleidomastoid muscles during the performance of efforts in extension, flexion, left and right lateral bending, and protraction. The EMD was extracted using the Teager-Kaiser Energy Operator. Reliability indices calculated for each muscle in each testing direction were: the difference in scores between the two testing sessions and corresponding 95% confidence intervals, the standard error of measurement (SEM) and intra-class correlation coefficients (ICC).

**Results:**

EMD values showed no evidence of systematic difference between the two testing sessions across all muscles and testing directions. The SEM for extension, flexion and lateral bending efforts ranged between 2.5 ms to 4.8 ms, indicating a good level of measurement precision. For protraction, SEM values were higher and considered to be imprecise for research and clinical purposes. ICC values for all muscles across all testing directions ranged from 0.23 to 0.79.

**Conclusion:**

EMD of selected neck muscles can be measured with sufficient precision for the assessment of neck muscle function in an athletic population in the majority of directions tested.

## Introduction

Concussions related to sport participation are a serious health problem, for which a multi-tiered approach has been recommended. This approach focuses on reducing both the risk for concussion as well as the severity of injury during a concussive event through improvement of protective equipment [1], modification of playing rules [2], emphasis on fair play [3], and development of comprehensive guidelines regarding return-to-play decision making [4]. Neck strengthening exercises have also been recommended as part of a comprehensive sport participation program to manage the risk for concussion [5-7]. This recommendation, however, is supported only by indirect evidence from studies that have used model simulations to show that, theoretically, increases in neck stiffness would achieve substantial reductions in the magnitude of head accelerations during collisions [7-9].

A first step toward translating the predictions of these simulations to the design of evidence-based strength training programs for concussion prevention is the ability to reliably measure relevant variables of neck muscle function. An athlete's ability to stiffen the neck in preparation for an upcoming collision is dependent in part on both relative situational awareness and the delay between onset of activity of relevant muscles and the onset of force production. Electromechanical delay (EMD) quantifies this latter aspect of the neuromuscular response and has been well documented for a variety of muscles in different types of contractions [e.g. [10-12]]. However, there is scarcity in information pertaining to EMD of the neck musculature during performance of maximal exertions, and no investigations have addressed the reproducibility of this measure in a between-day test-retest scheme.

The purpose of this study was to assess the reliability of EMD in subjects performing maximal voluntary contractions (MVCs) of the neck muscles in five different directions.

## Methods

### Subjects

Twenty-one healthy athletic males participated in this study (age: 21 (1.2) years, height 1.88 (0.07) m, weight 82.6 (5.4) kg, neck circumference 0.56 (0.02) m, and head circumference 0.38 (0.02) m). Subjects were involved in physical training 4 to 8 times per week at the university, national or elite competitive levels. None of these activities incorporated specific training of the neck musculature. Prior to testing, all subjects provided written informed consent and completed a self-report medical questionnaire to screen for specific exclusion criteria suggested by Sommerich et al. [13] when testing MVCs of the neck. All methods and procedures for this study were approved by the University Research Ethics Board.

### Instrumentation

**Figure 1:**
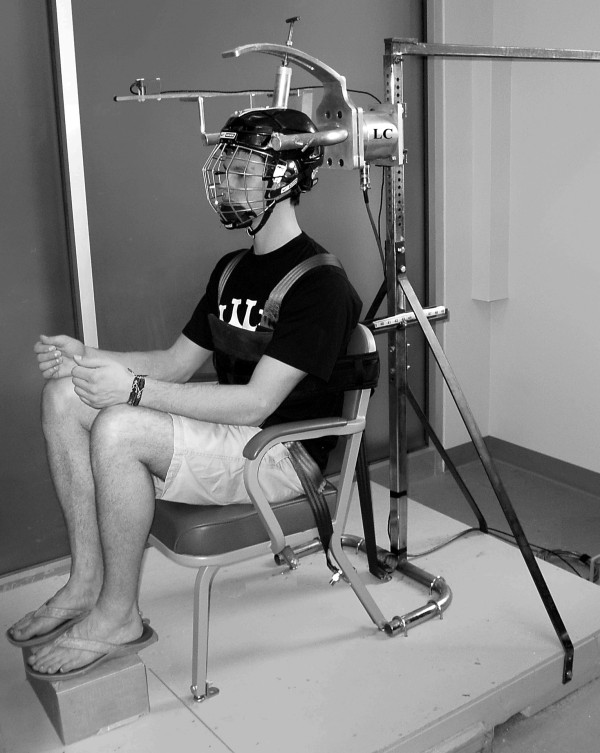
**Custom-built fixed-frame dynamometer**. the helmet restraint structure is appended to a 6 degree of freedom load cell (LC) for measurement of efforts exerted in different directions.

A custom built, fixed-frame static dynamometer was used to simultaneously record force and EMG measures during MVCs of the neck muscles (Figure 1). The device consists of a load cell coupled to a semi-spherical aluminum structure used for attachment of a hockey helmet incorporating a face mask (Bauer Nike, St. Jerome, Quebec, CA). The position of the helmet in the sagittal plane as well as the height of the load cell and the distance of the chair relative to the load cell can be adjusted to accommodate individual subject anthropometrics. Subjects' perform testing while seated and firmly restrained. Subjects-exerted neck muscle forces are recorded using six-degree-of-freedom load cell (MC5-2500, AMTI, Watertown, MA, USA). Which is interfaced with a multi-channel amplifier (Modular 600, frequency response 0-1 kHz, CMRR 110 dB at 60 Hz, input impedance 100 MΩ) (RDP Group™, Pottstown, PA, USA). The accuracy of the load cell factory-calibration specifications was verified prior to testing.

EMG data were recorded using a Bortec AMT-8 amplifier (frequency response 10 Hz to 1 kHz, CMRR < 115 dB at 60 Hz, input impedance > 1 GΩ, gain range 2000-5000) and sampled with the load cell using a common 16-bit Analog to Digital (A/D) converter (NI PCI-6036E, range of ± 5V) at 2048 Hz using custom-software written in Labview™ version 8.6 (National Instruments Inc., Austin, TX, USA).

### Procedures

Subjects completed two testing sessions seven to eight days apart. All tests were performed at the same time of day to control for diurnal effects. Prior to each testing session, subjects completed a series of warm-up exercises involving movement of the head and neck through full range of motion, passive stretching at end range, and self-resisted sub-maximal and maximal isometric excursions in the five directions of testing (extension, flexion, left and right lateral bending, and protraction).

**Figure 2:**
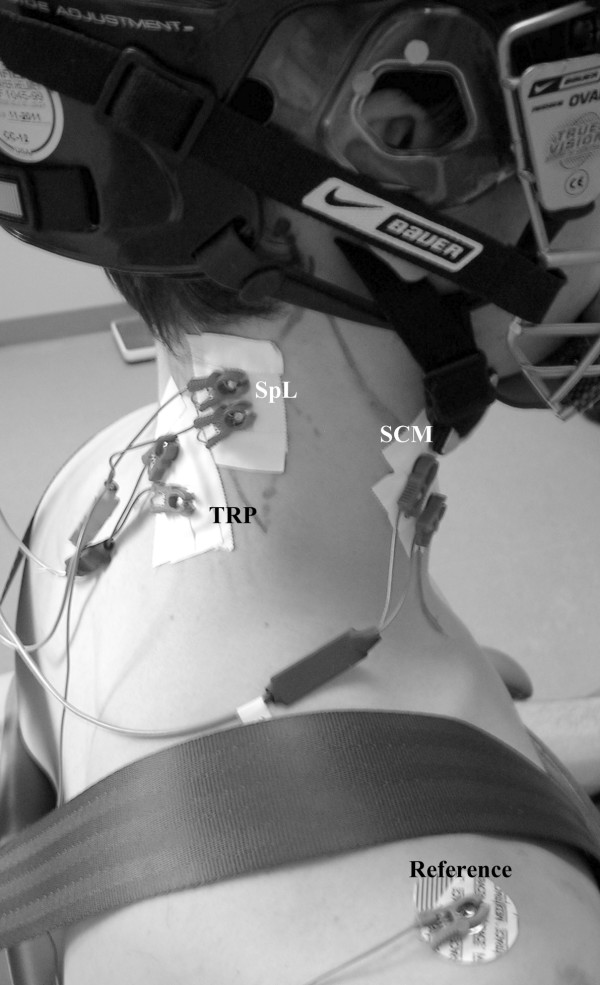
Surface EMG electrode placement sites for the right sternocleidomastoid (SCM), splenius capitis (SpL) and upper trapezius (TRP).

Surface EMG activity was recorded using bipolar self-adhesive, pre-gelled Ag-Ag/Cl electrodes with an inner diameter of 10 mm (Bortec Biomedical Ltd., Calgary, Alberta, CA) from the sternocleidomastoid (SCM), splenius capitis (SpL) and upper trapezius (TRP) muscles bilaterally (Figure 2). Prior to electrode placement, the subjects' skin was shaved, abraded with fine sand paper and cleansed with 70% isopropyl alcohol. For the SCM, the electrodes were placed along the sternal portion of the muscle, with the electrode centre 1/3 of the distance between the mastoid process and the sternal notch [14,15]. For SpL, the electrode centre was located at the intersection of the C7-ear line and the line of action of the SpL muscle that had been palpated during examiner-induced resistance to isometric exertions of the head in rotation [16]. For the TRP, the medial electrode was placed 2 cm lateral to the midpoint of the C4-C5 inter-spinous distance and oriented along the palpated anterior border of the trapezius, in line with the direction of the muscle fibers [17]. For SCM and TRP, the inter-electrode distance was 20 mm, while a 12 mm distance was used for the SpL in order to minimize the chance of overlapping adjacent muscles. A common reference electrode was placed on the right acromium process (pre-gelled, Ag-Ag/Cl, 10 mm inner diameter, Meditrace Model 135, Kendall, MA, USA). The electrodes were further secured to the skin using skin tape. Prior to recording, the electrodes were allowed to stabilize for 10 to 15 minutes, and tested with an ohmmeter to insure an electrode-skin impedance level of less than 10 kΩ.

Subjects were then fitted with the hockey helmet, seated and restrained in the device. The helmet was attached to the fixed frame and its position was adjusted to correspond to measurements of a self-determined, neutral posture of the head and neck recorded beforehand using a 3-Space Isotrak digitizer system (Polhemus Navigation Sciences, Colchester, VT, USA). Subjects were instructed to keep both hands on their thighs, and to place their feet on a cardboard box. The placement of the feet on the box enabled audible and visual identification of leg muscular contribution to force production, as pushing down on the box would collapse it and pushing to the sides would translate the box across the floor.

Subjects performed several sub-maximal practice trials within the testing apparatus in each direction in order to familiarize with the experimental task; once comfortable, subjects performed one or two MVC practice trials in each direction. For testing, subjects were instructed to develop force as fast as possible, and then to hold this force level until the end of the trial. Subjects performed four MVCs in each direction for a total of 20 trials. Each trial lasted four seconds in duration, with a 30 s rest period given between trials. When contribution by the lower extremities was detected, the trial was discontinued and repeated after a 30 s rest period. The order of directions during testing was randomized both within and between subjects. The same order of movement directions was used for the two testing sessions. Visual feedback on performance and consistent verbal encouragement were provided throughout each testing session. All procedures were administered by a single examiner.

### Signal Processing

**Figure 3:**
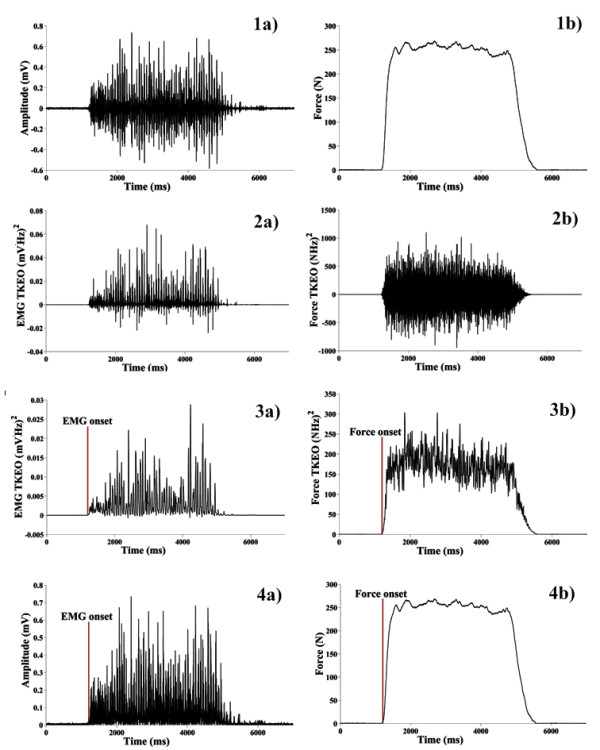
**Process of EMG (a) and force (b) onset detection**. 1a, b) Raw EMG and force signals. 2a, b) Teager Kaiser Energy Operator (TKEO) output for EMG and force signals. 3a, b) Full wave rectified and filtered TKEO outputs. Onset was detected at this stage using preset thresholds. 4a, b) Full-wave rectified EMG and raw force signals used to ascertain onset instances. The EMG signal and accompanying force curve depicted is from the right SpL of one of our participants during an extension effort.



Force and EMG signals were zero offset prior to processing. The first step in determination of EMD involved the computation of the Teager-Kaiser Energy Operator (TKEO) for both raw EMG [18,19] and force signals (Figure 3). The TKEO is a local energy measure for oscillating signals which is proportional to the signal's instantaneous amplitude and frequency [18-22]. In its discrete form, the TKEO(Ψ) value of a signal is given by [18-22]:

where *x *is the EMG or force signal and *n *is the sample number.

Li et al. [18] and Solnik et al. [19] have shown, using both simulated and real EMG signals, that application of the TKEO effectively suppresses baseline activity where the signals energy is 'low', relative to the time duration of muscle contraction where the energy of the signal is 'high'. This property of the TKEO is especially useful if the acquired EMG exhibits a low signal to noise ratio or a fluctuating baseline activity, which would bias the calculation of the reference value needed for onset detection when relying on common threshold-based methods [e.g. [23,24]]. In this investigation, we used TKEO for detection of onsets because we were concerned with potential false positive onset declaration resulting from heart muscle activity (ECG), a problem that has been observed by several authors in the recording of neck EMG [25,26]. In addition, it was also sometimes difficult to assess the onset of the muscle activity when a given muscle exhibited low levels of activity. Onset of force signals was also processed using the TKEO due to small fluctuations in force baseline activity resulting from small head stabilization movements prior to excursions.

Subsequent to TKEO calculations, EMG and force signals were full wave rectified and low pass filtered using a 2^nd ^order, zero phase shift, butterworth filter with a cutoff frequency of 50 Hz [24]. EMG and force onset thresholds were then set as the instant the signal exceeded 13 standard deviations above baseline levels for a period of 20 ms. All force and EMG onsets were verified visually by a single examiner on the original force and full wave rectified EMG signals. EMD was then calculated as the difference between the onset of EMG and the onset of force (units ms). Negative EMD values indicate that EMG preceded force onset, while positive values indicate that force onset preceded EMG activity onset.

### Statistical Analysis

All trials were reviewed, and the 3 trials with the highest rate of force development (e.g., the maximal value of the slope of the force-time curve) were selected. The average EMD values from these trials were used in the following analysis.

Normality of distribution for EMD values was assessed for all testing directions by visual inspection of histogram plots and by Shapiro-Wilks normality tests (p ≤ 05): all data met the normality requirements for parametric statistics. The difference in average scores between testing sessions (i.e. Day 2 score - Day 1 score) were computed along with the corresponding 95% confidence intervals to identify systematic bias [27]. The standard error of measurement (SEM) was used to determine measurement precision. SEM was calculated by dividing the standard deviation of the difference scores by the square root of two [27]. The smallest detectable difference value (SDD), used to determine the smallest change necessary for declaration of statistically significant differences between measurements from the two testing sessions, was calculated by multiplying the SEM by 2.77 [27], and as such utilized a 95% confidence level.

Retest correlation was assessed using intraclass correlation coefficients (ICCs), using the 3, k model [28]. This decision to use this type of model was done after examination of the confidence interval range of the between sessions difference scores, as recommended by Wier [29].

## Results

Table 1 summarizes the results obtained for the EMD: small differences in mean scores of less than 4.0 ms are evident between the two testing sessions across all muscles and testing directions. The corresponding 95% confidence intervals for all comparisons overlap zero, indicating that differences between test sessions are not statistically significant. Efforts exerted in extension were the most precise, with SEMs ranging from 2.5 ms to 4.0 ms, and corresponding SDD values ranging from 7.3 ms to 11.7 ms. ICC scores for extension ranged between 0.52 and 0.79.

**Table 1:** Electromechanical delay reliablity indices

	**Muscle**	**D1 Mean (SD)**	**D2 Mean (SD)**	**Diff (95% CI)**	**SEM (95% CI)**	**ICC_3,3 _(95% CI)**	**SDD**
**Ext**	R SpL	-31.0 (4.3)	-32.8 (6.8)	-1.8 (-3.9 to 0.4)	3.4 (2.6 to 4.8)	0.79 (0.49 to 0.91)	9.9
	R TRP	-37.4 (3.8)	-38.7 (2.7)	-1.3(-2.9 to 0.3)	2.5 (1.9 to 3.6)	0.59 (0.01 to 0.83)	7.3
	R SCM	3.3 (3.7)	3.0 (3.3)	-0.3 (-2.1 to 1.5)	2.8 (2.2 to 4.1)	0.52 (-0.19 to 0.80)	8.3
	L SpL	-33.1 (5.8)	-35.5 (6.1)	-2.4 (-4.9 to 0.2)	4.0 (3.0 to 5.7)	0.71 (0.29 to 0.88)	11.7
	L TRP	-38.3 (4.5)	-39.4 (4.5)	-1.1 (-3.0 to 0.9)	3.0 (2.3 to 4.3)	0.71 (0.29 to 0.88)	8.9
	L SCM	2.2 (5.5)	1.4 (5.0)	0.8 (-2.9 to 1.3)	3.3 (2.5 to 4.8)	0.74 (0.38 to 0.89)	9.8

**Flex**	R SpL	-32.8 (4.8)	-32.3 (4.2)	0.5 (-2.2 to 3.2)	4.2 (3.2 to 6.0)	0.23 (-0.88 to 0.68)	12.3
	R TRP	12.0(6.4)	13.9 (5.9)	1.8 (-0.9 to 4.6)	4.2 (5.2 to 6.1)	0.69 (0.25 to 0.87)	12.4
	R SCM	-70.6 (4.9)	-72.0 (4.4)	-1.5 (-4.4 to 1.1)	4.0 (3.0 to 5.7)	0.41(-0.44 to 0.76)	11.7
	L SpL	-30.2 (4.6)	-30.8 (4.8)	-0.6 (-2.7 to 1.6)	3.4 (2.6 to 4.9)	0.66 (0.17 to 0.86)	9.9
	L TRP	9.6 (4.4)	8.4 (5.3)	-1.2 (-3.5 to 1.1)	3.6 (2.7 to 5.1)	0.64 (0.11 to 0.85)	10.5
	L SCM	-69.8 (5.4)	-69.5 (5.0)	0.3 (-2.4 to 3.0)	4.2 (3.2 to 6.0)	0.52 (-0.17 to 0.80)	12.3

**Prot**	R SpL	-27.5 (9.9)	-27.9 (11.8)	-0.4 (-6.0 to 5.1)	8.6 (6.6 to 12.5)	0.54 (-0.12 to 0.86)	25.4
	R TRP	17.1 (10.8)	20.1 (12.5)	3.0 (-2.9 to 8.9)	9.2 (7.0 to 13.2)	0.77 (0.43 to 0.91)	27.0
	R SCM	-67.8 (13.9)	-66.4 (16.2)	1.4 (-6.3 to 9.2)	12.0 (9.2 to 17.4)	0.54 (-0.14 to 0.81)	35.5
	L SpL	-26.7 (12.3)	-27.9 (18.1)	-1.2 (-7.7 to 5.3)	10.1 (7.7 to 14.6)	0.73 (0.34 to 0.89)	29.8
	L TRP	18.2 (10.4)	18.0 (11.9)	-0.2 (-6.7 to 6.2)	10.0 (7.7 to 14.4)	0.32 (-0.66 to 0.72)	29.5
	L SCM	-68.1 (11.0)	-64.2 (13.6)	3.9 (-3.3 to 11.2)	11.3 (8.6 to 16.3)	0.31 (-0.68 to 0.72)	33.2

**LLB**	R SpL	18.8 (4.1)	19.5 (5.5)	0.7 (-1.9 to 3.4)	4.1 (3.1 to 5.9)	0.46 (-0.33 to 0.78)	12.0
	R TRP	-20.2 (4.9)	-19.8 (5.3)	0.4 (-2.2 to 2.9)	3.9 (3.0 to 5.6)	0.59 (-0.01 to 0.83)	11.5
	R SCM	0.7 (5.6)	1.3 (4.3)	0.5 (-2.1 to 3.1)	4.0 (3.1 to 5.8)	0.51 (-0.19 to 0.80)	11.9
	L SpL	-63.0 (6.0)	-62.2 (5.8)	0.8 (-2.0 to 3.6)	4.4 (3.4 to 6.3)	0.61 (0.05 to 0.84)	12.9
	L TRP	-43.8 (5.8)	-42.7 (4.3)	1.1 (-1.2 to 3.5)	3.7 (2.8 to 5.3)	0.64 (0.11 to 0.85)	10.9
	L SCM	-58.8 (6.0)	-56.4 (6.0)	2.5 (-0.2 to 5.1)	4.2 (3.2 to 6.0)	0.68 (0.22 to 0.87)	12.3

**RLB**	R SpL	-61.4 (5.2)	-59.6 (3.3)	1.8 (-0.6 to 4.2)	3.8 (2.9 to 5.5)	0.39 (-0.48 to 0.75)	11.1
	R TRP	-38.0 (4.6)	-39.7(5.3)	-1.7 (-4.0 to 0.6)	3.5 (2.7 to 5.1)	0.65 (0.15 to 0.86)	10.4
	R SCM	-62.9 (6.5)	-63.2 (6.3)	-0.3 (-3.4 to 2.8)	4.8 (3.6 to 6.9)	0.61 (0.05 to 0.84)	14.0
	L SpL	28.9 (6.7	29.8 (5.6)	1.0 (-1.7 to 3.6)	4.1 (3.2 to 6.0)	0.71 (0.28 to 0.89)	12.2
	L TRP	-18.5 (6.9)	-16.9 (6.1)	1.6 (-1.2 to 4.5)	4.4 (3.4 to 6.4)	0.70 (0.27 to 0.88)	13.0
	L SCM	2.3 (5.2)	1.4 (5.0)	-0.9 (-3.4 to 1.5)	3.8 (2.9 to 5.5)	0.60 (0.02 to 0.83)	11.3

**Ext **= Extension, **Flex **= Flexion, **Prot **= Protraction, **LLB **= Left lateral bending, **RLB **= Right lateral bending,

**SpL **= Splenius Capitis, **TRP **= Upper Trapezius, **SCM **= Sternocleidomastoid.

**D1 **= Day 1, **D2 **= Day 2, **SD **= Standard deviation, **Difference **= Difference between means,

**CI **= Confidence intervals, **ICC **= Intraclass correlation coefficient, **SDD **= Smallest detectable difference **(ms)**

Lateral bending efforts to both sides as well as flexion efforts resulted in slightly higher SEM scores, ranging between 3.4 ms and 4.8 ms. Naturally, the corresponding SDD values were larger for these testing directions, ranging from 9.9 ms to 14.0 ms. ICC scores for these directions are low to moderate, ranging between 0.23 and 0.71. Of note is that some corresponding confidence intervals were negative (e.g. right SpL, right SCM and left SCM in flexion).

Protraction elicited poor reliability for EMD, with SEM scores between 8.6 ms and 12.0 ms, and SDD values between 25.4 ms and 35.5 ms. ICC were low to moderate, ranging from 0.31 to 0.77, with some confidence intervals values being negative.

## Discussion

Measurement of neck neuromuscular functions using EMG could provide valuable information to evaluate the effects of interventional programs as well as possibly provide objective measures about readiness to return to play following a concussion injury. However, establishment of the between-day reliability of relevant measures is a prerequisite if they are to be used as part of assessment procedures.

With regards to the results obtained in this study, it should be first noted that no significant differences in EMD scores were observed between the two testing sessions across all muscles and directions. It has been previously recommended with respect to neck muscular testing that a familiarization session be performed, as participants are usually not accustomed to performance of maximal efforts [30,31]. However, our sample consisted of participants that were highly physically active, and we postulated that a well designed pre-testing familiarization and warm-up routine would suffice in order to eliminate any learning effect or apprehension in eliciting such maximal efforts. This is an important finding, as performing a familiarization session necessarily entails the allocation of resources and extra participant involvement, both of which can be limiting factors from a practical perspective. Even so, in different participant populations, such as those suffering from impairment, we certainly agree that a familiarization session prior to testing may be warranted.

Forming definitive statements regarding the acceptability of EMD measurement precision was difficult, as only a few researchers have reported upon EMD reliability indices [32-36], and even less have reported upon the long-term effects of interventional programs on the EMD [37-39]. None of these studies addressed the EMD of the neck muscles. In addition, these studies used dynamic exercises as the primary intervention, and in that respect, their results are not as relevant to the static muscle contraction mode employed in this study. We have been able to identify only one investigation that used an isometric-based interventional program and measured its influence on the EMD. Kubo et al [39] report an average decrease in EMD of 15.3 ms following a 12 week training program of the of the knee extensors. Given that the SDD values obtained in our investigation for all efforts, barring protraction, fall well within this improvement, and that large improvements may be expected from those untrained in specific muscular conditioning [40], the measurement precision of EMD is deemed to be acceptable for future clinical and research purposes, except in the direction of protraction. While the reasons for the poor reliability of EMD measured in protraction are not completely clear, it is possible that providing more practice in this specific direction during the pre-testing procedures could improve outcomes.

Whilst the degree of precision of the EMD seems to be acceptable for the majority of movement directions, the ICC values obtained are generally considered low to moderate. ICCs are usually reported as part of reliability assessment because they convey whether different individuals may be distinguishable from one another [14,29,41,42]. However, amongst the shortcomings of ICC is the fact that, if the participants' score range is homogenous, then the magnitude of between-subject variability may closely resemble the magnitude of the within-subject variability, ultimately yielding a low or even negative ICC ratio [14,41,42]. In this study, examination of the EMD standard deviation values suggests that subjects' score range was indeed narrow, thus being the determinant factor for the low ICC scores obtained. However, given the precision of measurements, the EMD values obtained may be used as reference values in subsequent investigations employed with subjects exhibiting similar physical characteristics [14,29,41,42].

## Conclusion

The results of this investigation suggest that EMD of neck muscles during isometric MVCs can be measured with an acceptable level of precision. In addition, the lack of difference scores between testing sessions suggests that in highly trained participants, a well-designed pre-testing protocol may suffice in order to eliminate apprehension or learning effects. Based on these results, we plan to utilize the EMD as an outcome measure in subsequent investigations involving conditioning of the neck musculature for sport participation, as well exploring whether this measure might be used to assess readiness to return-to-play following injury.

## Competing interests

The authors declare that they have no competing interests.

## Authors' contributions

SA participated in the building of the testing apparatus, designed the experimental protocol, developed the software acquisition and analysis programs, collected all data, performed the statistical analysis and drafted the manuscript. LP conceived the study, designed the apparatus, and assisted in drafting and revising of the manuscript. SVP assisted in study coordination and data collection. JMS assisted in design of the apparatus, drafting and revising of the manuscript, and together with LP provided guidance on all aspects of the study pertaining to their supervisory roles. All authors read and approved the final manuscript.
